# HIV-Related Stigma and Treatment Adherence Among Gay, Bisexual, and Other Men Who Have Sex with Men Who Use Crystal Meth in the Metropolitan Area of Mexico City

**DOI:** 10.1007/s10508-024-02816-6

**Published:** 2024-02-26

**Authors:** Leonardo Jiménez-Rivagorza, Ricardo Orozco, María Elena Medina-Mora, Claudia Rafful

**Affiliations:** 1 Faculty of Psychology, Universidad Nacional Autónoma de México, Circuito Ciudad Universitaria, 04510 Coyoacan, Mexico City, Mexico; 2 Center for Global Mental Health, National Institute of Psychiatry, Mexico City, Mexico

**Keywords:** Stigma, HIV, Treatment adherence, Men who have sex with men, Methamphetamine, Sexual orientation

## Abstract

Internationally, HIV-related stigma and crystal methamphetamine (meth) use have been described as barriers to treatment adherence among gay, bisexual, and other men who have sex with men (gbMSM). Crystal meth use has been increasing among gbMSM in the Metropolitan Area of Mexico City (MAMC). Therefore, this study aimed to determine the association between HIV-related stigma and HIV treatment adherence among gbMSM who use crystal meth in the MAMC. This study was undertaken as part of an exploratory study of crystal meth use in the MAMC. The data were collected from September to December 2021 through an encrypted online survey. Participants (*n* = 89) were gbMSM adults living with HIV who reported crystal meth use in the past month that were recruited through an online snowball sampling. The online survey included questions about HIV treatment adherence, sexual behaviors, the Alcohol, Smoking, and Substance Involved Screening Test, and the HIV-Related Stigma Mechanisms Scale. Logistic regression analyses assessed the association between HIV-related stigma and HIV treatment adherence. The multivariate logistic regression model showed that, controlling for health insurance [adjusted odds ratio (AOR) = 0.13; 95% confidence intervals (CI)  = 0.02–0.59] and educational level (AOR = 0.16; 95% CI = 0.02–0.88), non-adherence to HIV treatment was independently associated with higher HIV-related stigma (AOR = 1.06; 95% CI = 1.01–1.12). Public health policies must include HIV-related stigma and substance use in treating gbMSM with HIV.

**Supplementary Information:**

The online version contains supplementary material available at 10.1007/s10508-024-02816-6.

## Introduction

In 2022, there were 227,378 estimated persons living with HIV (PLWHIV) in Mexico (CENSIDA, 2023b). In the same year, Mexico City had an incidence rate of 13.7 per 100,000 persons, similar to the 13.5 national rate (CENSIDA, 2023b). Of the total number of persons diagnosed, 127,992 persons were enrolled in antiretroviral therapy (ART) by 2023 (CENSIDA, 2023a). Contrary to UNAIDS goals proposed in 2020 (UNAIDS, 2020), HIV incidence rates among the Mexican population have not decreased in recent years; there were 17,620 new cases in 2022 (CENSIDA, 2023b). According to the HIV cascade of care estimated in 2021, out of the total number of PLWHIV, 82% were diagnosed, out of which 91% were enrolled in ART and 88% of these were virally suppressed (Bravo-García et al., 2022). Moreover, HIV-related deaths remain an unsolved problem for Mexican public health institutions (CENSIDA, 2020); in 2019 there was an incidence mortality rate of 6.39 per 100,000 men, which represents 0.94% of the total deaths among men, an annual increase of 1.8% (Institute for Health Metrics & Evaluation, 2019).

According to the most recent nationally representative household addictions survey, there was an increase in substance use in Mexico City from 2011 to 2016 (2011 = 7.9% vs. 2016 = 10.3%), especially in cannabis (2011 = 6.7% vs. 2016 = 9.1%), cocaine (2011 = 2.6% vs. 2016 = 3.0%), and amphetamine-type stimulants (2011 = 0.5% vs. 2016 = 0.8%) (Instituto Nacional de Psiquiatría Ramón de la Fuente Muñiz et al., 2017). A national report on sexual diversity and substance use calculated a last year prevalence of 46% among gay, bisexual, and other men who have sex with men (gbMSM) (Baruch-Dominguez et al., 2015). As in other contexts (Lafortune et al., 2021; Rosińska et al., 2018), in Mexico polysubstance use among gbMSM has been associated with living with HIV (Algarin et al., 2023).

Previous research on the Mexican population has shown factors negatively affect HIV treatment adherence (Katz et al., 2013). Some of them are risky sexual behaviors, HIV-related stigma, and crystal methamphetamine (meth) use (Almanza-Avendaño & Gómez San Luis, 2017; Katz et al., 2013; Martín-Sánchez et al., 2002; Sierra Madero et al., 2015).

There are several theoretical proposals for defining HIV-related stigma (Goffman, 2006; Katz, 2014; Scambler & Hopkins, 1986). Earnshaw and Chaudoir (2009) proposed three different HIV-related stigma mechanisms due to the differential impact each has on the health of PLWHIV: internalized (i.e., self-negative vision), anticipated (i.e., the negative social perception that causes PLWHIV to expect receiving stigma), and enacted stigma (i.e., previous experiences of stigma). HIV-related stigma has been associated with HIV testing avoidance, not disclosing HIV-positive status, and stopping ART, thus affecting adherence to HIV treatment (Caldera-Guzmán & Pacheco-Zavala, 2020; Camacho et al., 2020; Rao et al., 2007; Restrepo Pineda, 2016).

Crystal meth use has been associated with an increased risk of HIV seroconversion and risky sexual behaviors (Hoenigl et al., 2016; Plankey et al., 2007), especially among gbMSM and other sexual minorities (Grov et al., 2020). Previous research on gbMSM living with HIV has reported that HIV-related stigma and the use of alcohol and stimulants are associated with symptoms of psychiatric disorders, risky sexual behaviors, and lower HIV treatment adherence (Earnshaw et al., 2013; Javanbakht et al., 2020; Sun et al., 2020).

Prior studies that have approached HIV-related stigma and treatment adherence in the Mexican population have been conducted from a qualitative perspective (Bermúdez-Román et al., 2015; Flores & Almanza, 2013; Saucedo Pahua et al., 2018) or have only focused on perceived or anticipated stigma (Ortiz-Hernández et al., 2021; Sierra Madero et al., 2015). To our knowledge, studies on HIV treatment adherence among persons who use methamphetamine are scarce. Therefore, we aimed to determine the association between HIV-related stigma and non-adherence to HIV treatment among cisgender (cis) gbMSM who use crystal meth in the MAMC.

## Method

### Participants and Procedure

These analyses were undertaken as part of a cross-sectional exploratory study on crystal meth use among adults living in the MAMC. Given the pandemic of COVID-19 and based on other health science studies (Rafful et al., 2022; Saberi, 2020), participants were recruited from September to December 2021 through a web-based snowball sampling. The snowball was initiated through nongovernmental organizations (NGOs) and a public clinic in Mexico City that provides HIV-related care. Assistance from stakeholders consisted of allowing the research team to place flyers in their facilities. These flyers included a QR code that led to the online survey. In addition, NGOs also promoted the research study on their social media and websites. Participants were also invited through Grindr and Hornet, which are geolocation dating apps targeted at gbMSM.

The inclusion criteria for the main project (*n* = 184) included being at least 18 years old, living in the MAMC, not being in treatment for substance use at the time of the study, and reporting crystal meth use in the past 3 months. For the present study, we analyzed the subsample (*n* = 89) of participants who met three criteria: self-identified as cis men (“How do you identify yourself?"). Answer options were “cisgender man,” “cisgender woman,” “transgender (trans) man,” and “trans woman,” and an open-ended response in case participants had a different sexual identity), reported having sex with men in the last year (“How many sexual partners have you had in the last year?”). Answer options included specific information on men, women, trans men, and trans women as sexual partners), and had a positive HIV diagnosis (“Have you ever been diagnosed with HIV in your life?”); only those who answered “yes” were included in the study regardless of the time living with HIV.

Persons who agreed to participate answered an online questionnaire that was available in Survio (2022), which is a platform that encrypts data and a secure site that guarantees the confidentiality of the information.

### Measures

Non-adherence to HIV treatment was assessed according to Mexican national guidelines (CENSIDA, 2021a), whereby the participants were asked about being on ART at the time of the study (yes/no), the frequency of attendance at their follow-up medical appointments related to HIV (never/few of the time/some of the time/most of the time/always), and taking ART as prescribed in the last 3 months (never/few of the time/some of the time/most of the time/always). Thus, participants who reported (1) being enrolled on ART at the time of the study, (2) taking their ART, and (3) attending their follow-up medical appointments “always” or “most of the time” were identified as persons with adherence to HIV treatment. Participants who did not meet any of the three conditions were classified as participants with inconsistent adherence. Further on, we will refer to this group as participants without adherence. Additionally, participants were asked about their reasons for always taking or not ART (Varela Arévalo et al., 2008).

HIV-related stigma was assessed with the HIV Stigma Mechanisms Scale (HIV-SMS) (Earnshaw et al., 2013), which is based on the HIV-related stigma mechanisms framework (Earnshaw & Chaudoir, 2009), and validated for the Mexican population reporting adequate internal consistency and reliability (Ω = 0.86) (López et al., 2023a). The HIV-SMS consists of 3 subscales: internalized, anticipated, and enacted stigma. The total scale is assessed through 18 Likert-scale items ranging from 1 (never/ strongly disagree/ very unlikely, respectively) to 5 (always/ strongly agree/ very likely, respectively). This scale provides a score for each mechanism and a total score based on the addition of the 3 subscales.

Sociodemographic variables included in this study were age (continuous), sexual orientation (gay, bisexual, heterosexual, other), relationship status (in a relationship, single), health insurance (insured, uninsured), main income source (no income, scholarship/government assistance, drug dealing, partner/friends/family, sex work), educational attainment (high school or less, undergraduate studies or more), and monthly average income asked in Mexican pesos according to minimum wage rates in 2020 (< USD$49.3, USD$49.3–USD$133.6, USD$133.7–USD$267.2, USD$267.3–USD$400.9, USD$400.9–USD$668.2, > USD$668.2) (Instituto Nacional de Estadística y Geografía, 2022).

Past-year sexual behaviors included the number of sexual partners in the past year (continuous) and having had condomless sex with persons with a known or suspected STI (HIV, HCV, syphilis, or other; yes/ no). In addition, participants were asked about crystal meth use before or during sexual intercourse and whether they had received or given crystal meth in exchange for a sexual encounter assessed through Likert-scale questions ranging from 1 (never) to 5 (always) that were merged into “sometimes or less” and “most of the time or always.”

Clinical HIV characteristics included were the years since diagnosis (continuous), years on ART (continuous), and the evolution of HIV to AIDS (yes/ no).

The risk associated with substance use was assessed using the Alcohol, Smoking, and Substance Involvement Screening Test (ASSIST) version 3.1 (OMS, 2011). This screening tool provides information for health care providers and researchers on substance use patterns, and it is widely used in clinical and epidemiological studies (Lee et al., 2023). ASSIST scores are categorized as low, moderate, or high risk associated with each type of substance in the past 3-months. A low-risk use of alcohol corresponds to a score of 0–10 and 0–3 for all other substances, a score of 11–26 indicates a moderate-risk use of alcohol and 4–26 for all other substances, and a high-risk use for all substances corresponds to a score of > 27 (OMS, 2011). The ASSIST reliability among the Mexican population varies between 0.80 and 0.87 (Tiburcio Sainz et al., 2016). Due to the high prevalence of substance use in the sample, for the analyses, we dichotomized the variable into “low and medium risk” and “high risk.”

### Data Analyses

We used nonparametric measures of central tendency (i.e., median [Mdn] and interquartile range [IQR]) and summary (i.e., frequency and percentage) to analyze the sociodemographic variables and the distribution of the independent variables in the sample. Logistic regression models were performed to identify the association between HIV-related stigma and non-adherence to HIV treatment. (This outcome variable was coded as 0 = adherence and 1 = non-adherence.) This analysis was repeated to identify the association between non-adherence to HIV treatment and each independent variable. Multiple models were used to identify the association between HIV-related stigma and non-adherence to HIV treatment controlling for the simultaneous effect of other independent variables. All the variables that were associated with non-adherence to HIV treatment at *p* < 0.10 in the bivariate models were included in a correlation matrix (Supplementary Material) to assess for collinearity. Subsequently, variables with correlations *r* > 0.5 and *p*-values < 0.05 were excluded from the final model (Ambrosius, 2007). The final model, based on the forward stepwise selection, was performed by calculating the pseudo-R^2^ (McFadden) and the Akaike information criteria (AIC) measures. All statistical analyses were performed in RStudio statistical software 4.1.2 version (R Core Team, 2023).

## Results

### Sociodemographic Characteristics

A sample of 89 gbMSM that used crystal meth in the last 3 months with a median age of 34 years old (IQR = 30–38) reported living with HIV and provided data on HIV treatment adherence (Table 1); most participants reported HIV treatment adherence (82.02%), self-identified as gay (95.6%), not being in a relationship (83.52%), having high educational attainment (81.32%), a monthly income greater than $400.9 (60.23%), having a formal job as the main income source (85.71%), and having health insurance (86.35%). Health insurance and educational level were significantly negatively associated with non-adherence to treatment.

**Table 1:** Sociodemographic characteristics and bivariate associations with HIV treatment non-adherence, among men who have sex with men living with HIV that use crystal meth in the Metropolitan Area of Mexico City 2021 (*n* = 89)

	Persons with adherence		Persons with no-adherence		Total (n = 89)		OR	95% CI		
	n/Mdn	%/ IQR	n/Mdn	%/ IQR	n/Mdn	%/ IQR				
Age	32	30–38	34	30–39.50	34	30–38	1.00	0.92		1.08
*Sexual orientation*										
Bisexual	3	4.11	1	6.25	4	4.40	–	–		–
Gay	70	95.89	15	93.75	87	95.60				
*Relationship status*										
In a relationship	13	17.81	2	12.50	15	16.49	1.00			
Single	60	82.19	14	87.5	76	83.52	1.52	0.36		10.4
*Educational level*										
High school or less	8	10.96	9	56.25	17	18.68	**1.00**			
Completed undergraduate level	65	89.05	7	43.75	74	81.32	**0.10**	**0.03**		**0.32**
*Monthly income*										
$400.9 or less	27	30.04	7	46.67	35	36.78	1.00			
More than $400.9	44	61.47	8	53.34	53	60.23	0.70	0.23		2.21
*Main income source*										
No income	3	4.11	0	0	3	3.30	–	–		–
Formal job	66	90.41	10	62.50	78	85.71				
Scholarship/government assistance	0	0	1	6.25	1	1.10				
Selling drugs	0	0	1	6.25	1	1.10				
Partner/friends/family	3	4.11	2	12.50	5	5.49				
Sex worker	1	1.37	2	12.50	3	3.30				
*Health insurance*										
None	12	16.67	8	53.33	20	22.73	**1.00**			
Any	60	83.34	7	46.67	68	86.35	**0.18**	**0.05**		**0.57**

Significance at* p* < 0.05 in bold.* Mdn* Median, *IQR* Interquartile range,* OR* Odds ratio,* CI* Confidence interval

Bivariate logistic regressions of the variables with a frequency of less than 5 are not reported.

### HIV-Related Variables

Participants without adherence reported statistically significant higher levels of total HIV-related stigma (Mdn = 45; IQR = 35.35–63.75 *vs.* Mdn = 29; IQR = 21–38), enacted stigma (Mdn = 7.50; IQR = 6–16.50 vs. Mdn = 6; IQR = 4–7), internalized stigma (Mdn = 16; IQR = 6–6.25 vs. Mdn = 10; IQR = 6–17.50), and anticipated stigma (Mdn = 15; IQR = 12.25–25.50 *vs.* Mdn = 11; IQR = 6–14) than their counterparts (Table 2).

**Table 2:** Descriptive statistics of HIV-related stigma and clinical variables and bivariate associations with HIV treatment non-adherence among men who have sex with men living with HIV that use crystal meth in the Metropolitan Area of Mexico City 2021 (*n* = 89)

	Persons with adherence		Persons with no-adherence		Total		OR	95% CI	
	n/Mdn	%/ IQR	n/Mdn	%/ IQR	n/Mdn	%/ IQR			
Total stigma	29	21–38	45	35.25–63.75	31	21–39	**1.07**	**1.03**	**1.11**
Enacted stigma	6	4–7	7.5	6–16.50	6	4.5–8	**1.17**	**1.06**	**1.31**
Internalized stigma	10	6–17.50	16	6–25.50	11	6–18	**1.08**	**1.00**	**1.16**
Anticipated stigma	11	6–14	15	12.25–25.50	12	7–15	**1.15**	**1.06**	**1.27**
Years since HIV-positive diagnosis	6	3–9	6	3–7	6	3–9	0.97	0.84	1.08
*Evolution to AIDS*									
No	65	90.28	10	71.43	76	86.36	1.00		
Yes	7	9.72	4	28.57	12	13.64	3.71	0.85	14.83
*ART at the time of study*									
No	0	0	8	50	8	8.89	–	–	–
Yes	73	100	8	50	82	91.11			
Years on ART	5	2.93–7.25	7	4.25–80	5	3–7	1.05	0.89	1.21
*Attendance of HIV-related medical appointments*^a^									
Never/sometimes	0	0	6	62.50	5	6.17	–	–	–
Most of the time/all the time	73	100	3	37.50	76	93.83			
*ART taking frequency*^a^									
Never/sometimes	0	0	7	87.50	7	8.54	–	–	–
Most of the time/ all the time	73	100	1	12.50	75	91.47			
*Reasons for not taking ART all the time*^a^									
Avoiding side effects	2	4.44	0	0	2	4.44	–	–	–
Not wanting to take ART	4	8.89	2	4.44	6	13.33			
Lack of trust in ART benefits	0	0	1	2.22	1	2.22			
Difficulties keeping schedule	2	4.44	2	4.44	4	8.89			
Financial barriers	0	0	1	2.22	1	2.22			
Forgetting to take the doses	22	48.89	3	6.67	25	55.56			
ART was unavailable	3	6.67	2	4.44	5	11.11			
*Reasons for taking ART all the time*^a^									
To experience the benefits	15	32.61	–	–	15	32.61	–	–	–
The medical staff asked me to take it	6	13.04	–	–	6	13.04			
For close persons	10	21.74	–	–	10	21.74			
Wants to stay healthy	40	86.96	–	–	40	86.96			
Other	2	4.35	–	–	2	4.35			

^a^Last 3 months. Significance at *p* < 0.05 in bold. “Mdn”= median, “IQR”= interquartile range, “OR”= Odds ratio, “CI”= Confidence interval. Bivariate logistic regressions of the variables with a frequency of less than 5 are not reported. Some percentage data do not add up to 100 because participants could choose more than one option. Bivariate logistic regressions of the variables related to treatment adherence or with a frequency of less than 5 are not reported

Overall, 6 years was the Mdn time with a positive diagnosis of HIV (persons with adherence IQR = 3–9; persons without adherence IQR = 3–7). Additionally, 9.72% of persons with adherence and 28.57% of participants without adherence reported that their HIV diagnosis had progressed to AIDS. Most participants were on ART at the time of the study (91.11%). Persons with adherence reported fewer years on ART than participants without adherence (Mdn = 5 *vs.* Mdn = 7); however, this difference was not statistically significant. Of those who reported currently being on ART, 93.83% attended their medical appointments most of the time or all the time. In contrast, 62.50% of participants without adherence reported having attended their medical appointments “never” to “a few times.” Regarding ART intake, all participants with adherence reported taking it most of the time or always, while 12.50% of persons without adherence did it most of the time. Of those who reported not always taking ART, reported the following reasons: 4.44% reported avoiding side effects, 13.33% not wanting to take ART, 2.22% lack of trust in ART benefits, 8.89% difficulties keeping a schedule, 2.22% financial barriers, 55.56% forgetting taking the doses, and 11.11% reported ART was unavailable. Among the reasons for always taking ART, most reported wanting to stay healthy as the reason for treatment adherence (86.96%), 32.61% reported experiencing the benefits of the prescription, 13.04% were following recommendations from health professionals, 21.74% did it because persons close to them asked them to take it, 4.35% reported not wanting to die of AIDS, and 4.35% not wanting to be hospitalized again.

### Sexual Behaviors

Regarding condom use, most of the participants have had condomless sex in the last year (participants with adherence = 90.41% *vs.* participants without adherence = 100%) (Table 3). Most reported condomless sex with another PLWHIV (participants with adherence = 93.94% *vs.* participants without adherence = 86.67%). Additionally, around a quarter of the sample reported having had condomless sex with someone known or suspected of living with HCV (participants with adherence = 21.21% *vs.* participants without adherence = 25%), syphilis (participants with adherence = 31.82% *vs.* participants without adherence = 25%), or another STI (participants with adherence = 26.15% *vs.* participants without adherence = 37.50%); there were no statistically significant differences by group. All participants reported only cis men as sexual partners, with a lower number of partners among persons with adherence than those without adherence (Mdn = 20 *vs.* Mdn = 50).

**Table 3:** Sexual behaviors, substance use, and bivariate associations with HIV treatment non-adherence among men who have sex with men living with HIV that use crystal meth in the Metropolitan Area of Mexico City 2021 (*n* = 89)

	Persons with adherence			Persons with no-adherence		Total		OR	95% CI	
	n/ Mdn		%/ IQR	n/ Mdn	%/ IQR	n/ Mdn	%/ IQR			
*Condomless sex*^a^										
No	7		9.59	0	0	7	7.69	–	–	–
Yes	66		90.41	16	100	84	92.30			
*Condomless sex with a person possibly living with HIV*^a^										
No	4		6.06	2	13.33	6	7.23	–	–	–
Yes	62		93.94	13	86.67	77	92.77			
*Condomless sex with a person possibly living with HCV*^a^										
No	52		78.79	12	75	66	78.57	–	–	–
Yes	14		21.21	4	25	18	21.43			
*Condomless sex with a person possibly living with syphilis*^a^										
No	45		68.18	12	75	57	67.86	–	–	-
Yes	21		31.82	4	25	27	32.14			
*Condomless sex with a person possibly living with another sexually transmitted disease*^a^										
No	48		73.85	10	62.50	60	77.29	1.00		
Yes	17		26.15	6	37.50	23	22.71	1.70	0.51	5.30
*Number of sexual partners*^a^										
Cis men	20		6–50	50	18.75–112.50	25	7–65	1.00	1.00	1.01
*Use of crystal meth during sexual intercourse*^a^										
Never/sometimes	28		38.36	4	25	32	35.16	1.00		
Most of time/all the time	45		61.65	12	75	57	64.84	1.87	0.58	7.19
*Receiving crystal meth in exchange for a sexual encounter*^a^										
Never/sometimes	71		97.26	11	68.75	84	92.31	1.00		
Most of the time/all the time	2		2.74	5	31.25	7	7.69	**16.14**	**3.08**	**123.00**
*Giving crystal meth in exchange for a sexual encounter*^a^										
Never/sometimes	66		90.41	11	73.34	79	87.77	1.00		
Most of the time/all the time	7		9.59	4	26.67	11	12.22	3.43	0.79	13.48
*Substance use score*^b^										
Crystal meth (*n* = 87)	20.5		13.25–26	26	16.50–29.50	22	13.50–27	1.04	0.98	1.13
Tobacco (*n* = 41)	12		8–17	8	5–19	12	8–17	0.99	0.88	1.10
Alcohol (*n* = 66)	4		2–12	4.5	2–7.25	4	2–90.75	0.93	0.77	1.05
Cannabis (*n* = 41)	8		4–15	4	2–13.50	7	2–15	0.96	0.86	1.06
Cocaine (*n* = 23)	3		2–12	17	13–19	5	2–16.50	**1.15**	**1.02**	**1.39**
Amphetamine-type stimulants (*n* = 10)	4		2–8	9	5.5–18.50	5	2–90.75	1.06	0.92	1.25
Sedatives or sleeping pills (*n* = 17)	7.5		3.50–11.75	5	3.5–7.50	6	3–11	0.90	0.61	1.07
Inhalants (*n* = 5)	5		4.25–7.50	32	32–32	5	5–15	14.53	0	0
Hallucinogens (*n* = 7)	2		2–4.25	2.5	2.25–2.75	2	2–5	0.79	0	1.41
Opiates (*n* = 3)	3		2.5–4.50	0	0	3	2.50–4.50	–	–	–

^a^Past-year

^b^ASSIST score among those who reported past-3 months of use per substance. Low risk 0-10 for alcohol and 0–3 for all other substances, moderate risk 11–26 for alcohol and 4–26 for all other substances, high risk 27+ for all substances. Significance at* p* < 0.05 in bold. “Mdn”= median, “IQR”= interquartile range, “OR”= Odds ratio, “CI”= Confidence interval. Bivariate logistic regressions of the variables with a frequency of less than 5 are not reported

Concerning crystal meth in sexual intercourse, compared to persons with adherence, those without adherence reported higher crystal meth use during sexual intercourse (75% vs. 61.65%;) as well as receiving (31.35% vs. 2.74%) and giving (26.67% vs. 9.59%) crystal meth in exchange of a sexual encounter. Only the differences regarding giving and receiving crystal meth were statistically significant.

### Risks Associated with Substance Use

Compared to participants with adherence to HIV treatment, persons without adherence had significantly higher risky use of cocaine [Mdn = 17 (moderate risk); IQR = 13–19 vs. Mdn = 3 (low risk); IQR = 2–12] (Table 3). Also, higher but not significantly different, were the moderate risk of crystal meth (Mdn = 26; IQR = 16.50–29.50 vs. Mdn = 20.50; IQR = 13.30–26), low-risk alcohol use (Mdn = 4.50; IQR = 2–7.30 vs. Mdn = 4; IQR = 2–12), low-risk amphetamine use (Mdn = 9; IQR = 5.5–18.50 vs. Mdn = 4; IQR = 2–8), and low-risk hallucinogens use (Mdn = 2.50; IQR = 2.30–2.80 vs. Mdn = 2; IQR = 2–4.30). Inhalant use was low and high risk, respectively (Mdn = 5; IQR = 4.3–7.50 vs. Mdn = 32; IQR = 32–32). In contrast, compared to persons with adherence, those without adherence had slightly lower risk in tobacco (Mdn = 8; IQR = 5–19 vs. Mdn = 12; IQR = 8–17), cannabis (Mdn = 4; IQR = 2–13.50 vs. Mdn = 8; IQR = 4–15), amphetamines (Mdn = 9; IQR = 5.5–18.50 vs. Mdn = 4; IQR = 2–8), sedatives (Mdn = 5; IQR = 3.50–7.50 vs. Mdn = 7.50; IQR = 3.5–11.80), and opiates (no use *vs.* Mdn = 3; IQR = 2.50–4.50).

### Factors Associated with HIV Treatment Adherence

Bivariate analyses showed that non-adherence to HIV treatment was associated with total HIV-related stigma [Odds Ratio (OR) = 1.07; 95% Confidence Intervals (CI) = 1.03–1.11], enacted (OR = 1.17; 95% CI = 1.06–1.31), internalized (OR = 1.08; 95% CI = 1.00–1.16), and anticipated (OR = 1.15; 95% CI = 1.06–1.27) mechanisms, the evolution of HIV diagnosis to AIDS (OR = 3.71; 95% CI = 0.85–14.83), having received (OR = 16.14; 95% CI = 3.08–123.00) and given (OR = 3.43; 95% CI = 0.79–13.46) crystal meth in exchange of a sexual encounter, and the risk associated with cocaine use (OR = 1.15; 95% CI = 1.03–1.40) (Table 4). On the other hand, the variables that lowered the odds of non-adherence were having health insurance (OR = 0.18; 95% CI = 0.05–0.57) and having completed undergraduate studies or more (OR = 0.10; 95% CI = 0.03–0.32). We found collinearity between the three HIV-related stigma mechanisms, the total HIV-related stigma, and cocaine use (Supplementary Material). Hence, we discarded the three HIV-related stigma mechanisms (in favor of the total score) and cocaine substance use to perform a set of multivariate regression models. The final model, which included total stigma, health insurance, and educational level, showed an explanatory variance of treatment adherence of pseudo-*R*^2^ = 0.38 and goodness of fit (AIC = 52.66). Controlling for health insurance [adjusted odds ratio (AOR) = 0.13; 95% CI = 0.02–0.59] and educational level (AOR = 0.16; 95% CI = 0.02–0.59), non-adherence to HIV treatment was independently associated with higher HIV-related stigma (AOR = 1.06; 95% CI = 1.01–1.12).

**Table 4:** Bivariate logistic regression models and final multivariate logistic regression model of variables associated with non-adherence to HIV treatment

		OR	95% CI			AOR	95% CI		
*HIV-related stigma*									
Total stigma		**1.07**	**1.03**		**1.11**	**1.06**	**1.01**		**1.12**
Enacted stigma		**1.17**	**1.06**		**1.31**				
Internalized stigma		**1.08**	**1.00**		**1.16**				
Anticipated stigma		**1.15**	**1.06**		**1.27**				
*Health insurance*									
None		1.00				1.00			
Public/private insurance		**0.18**	**0.05**		**0.57**	**0.13**	**0.02**		**0.59**
*Educational level*									
< High school		1.00				1.00			
> Undergraduate		**0.10**	**0.03**		**0.32**	**0.16**	**0.02**		**0.88**
Evolution to AIDS		3.71	0.85		14.83				
*Receiving crystal meth in exchange for a sexual encounter*^a^									
Never/sometimes		1.00							
Most of time/all the time		**16.14**	**3.08**		**123.00**				
*Giving crystal meth in exchange for a sexual encounter*^a^									
Never/sometimes		1.00							
Most of time/all the time		3.43	0.79		13.46				
Cocaine use		**1.15**	**1.03**		**1.40**				
Rx^2^						0.38			
AIC						52.66			

^a^Past year. Significance at p < 0.05 in bold. “OR”= Odds ratio, “CI”= Confidence interval, “AOR”= Adjusted odds ratio, “Rx2“= Pseudo-R2, “AIC”= Akaike's information criterion. The value of Rx2 was obtained from the McFadden measure

## Discussion

We aimed to identify the relationship between HIV-related stigma and non-adherence to HIV treatment among gbMSM who use crystal meth in the MAMC. We found that HIV-related stigma negatively affects treatment adherence, while educational attainment and having health insurance increased the odds of treatment adherence. Most participants were identified as persons with adherence to HIV treatment, which could be due to the sampling strategy. Nevertheless, previous studies in Mexico conducted with persons with job-related and public health insurance that offer ART have reported levels of HIV treatment adherence higher than 76%, similar to the proportion observed in this study (Hernández-Gómez et al., 2013; Pérez-Salgado et al., 2015).

Turan's et al.’s (2017a, 2017b) adaptation of Meyer’s (1995) minority stress model suggests that the impact of HIV-related stigma on HIV treatment adherence is mediated by several factors such as substance use and HIV status disclosure. Previous research in gbMSM from Mexico City showed that those participants who concealed their seropositive status to people close to them had a lower ART adherence, so PLWHIV may decide not to disclose their HIV status or follow their HIV treatment in front of people they believe will reject them (Ortiz-Hernández et al., 2021). Within the possible range of subscale values (López et al., 2023b), the study sample obtained a low score of stigma. This should be interpreted with caution because most of the participants had a formal job, and a higher level of education and income than the population average (Instituto Nacional de Estadística y Geografía, 2022). PLWHIV who use stimulants face an intersectionality of stigmas in which socioeconomic status may affect social interactions and, may mediate the loss of social status (Do et al., 2021; Lim et al., 2013). Consequently, the high socioeconomic status of this sample could explain the low expected, experienced, and internalized stigma reported in this study, as described in other samples across settings (Meyers-Pantele et al., 2022; Turan et al., 2017a, 2017b). Internalized stigma may act as an additional barrier in the dissemination of serological status to close people because this mechanism encourages self-exclusion from interpersonal relationships, distorts the perception of social support, and increases attachment-related anxiety (Turan et al., 2019; USAID, 2006). Regarding enacted stigma, some authors have reported that its association with treatment adherence is mediated by self-concept, depressive symptoms, and the risk associated with substance use (Earnshaw et al., 2020; Valenzuela et al., 2015). In addition, Algarin et al. (2020) found that the enacted HIV-related stigma and medium–high risk associated with substance use were related to non-adherence and non-suppression of viral load.

We cannot compare substance use prevalence with that of the general population considering that the sample was comprised of persons who used crystal meth in the past 3 months. Regarding the risk associated with substance use, we found a statistically significant association between non-adherence to HIV treatment and cocaine use. However, it was not further analyzed because of the collinearity between cocaine use and the main independent variable (i.e., stigma). We also found that persons without adherence reported slightly higher risk associated with crystal meth use than participants with adherence. Previous studies in gbMSM living with HIV suggest that harmful methamphetamine use affects the central nervous system, specifically in attention and verbal memory, which are important executive functions for treatment adherence (Lai et al., 2020; Wright et al., 2011). Additionally, it has been reported that crystal meth and cocaine use disorders had differential effects on adherence to HIV treatment due to the strong impact that stimulants have on the psychosocial environment of PLWHIV (Hinkin et al., 2007). Further analyses with a larger sample size are needed to better understand the association between stimulant use and non-adherence to HIV treatment in this setting.

The association between educational level and adherence to treatment found in this study has been previously reported; lower education may hinder understanding medical indications for taking ART (Pérez Bastán & Viana Castaño, 2020). However, studies of other chronic diseases have reported that people with primary-level studies had an adequate understanding of the disease (Gómez-Encino et al., 2015). Therefore, these results suggest that the impact of schooling on adherence to HIV treatment may be due to other variables such as the empowerment that PLWHIV may have about their condition (Veliz-Rojas et al., 2015), which could decrease their active participation when choosing a treatment to suit their needs.

Contrary to previous research (Kalichman et al., 2010), we did not find a significant association between condom use and sexual partners and non-adherence to HIV treatment. Possibly, this is because living with HIV and crystal meth use per se have been described as strong predictors of inconsistent condom use and having multiple partners regardless of adherence (McFarland et al., 2012). On the other hand, most participants reported having condomless sex with another PLWHIV but not with someone who had another STI. This alludes to the fact that the seroclassification of the participants may be related to considering risky having sex with a person who has syphilis or HCV but not with another HIV-positive person, in terms of not contemplating possible reinfections and its consequences (Villa-Rueda et al., 2021). These findings show the challenges that health professionals may face. Qualitative studies among persons who use crystal meth in high-income settings have also shown that they tend to prefer not to use condoms, but to adopt other self-care strategies such as adherence to PrEP programs (Closson et al., 2018; Drysdale et al., 2021), which would be a more plausible alternative to prevent new cases of HIV in persons who use crystal meth. PrEP is currently provided at public clinics for gbMSM and other key populations in Mexico City, but not across the country. More policy implementation is needed nationally.

Contrary to most studies, we did not find an association between crystal meth use during sexual intercourse and non-adherence to HIV treatment. However, this lack of association has been reported in some other studies (e.g., O’Halloran et al., 2019). In our study population, we hypothesize that this might be due to the incorporation of planned routines in which medication administration occurs in between substance use sessions, or by the prioritization of treatment adherence over substance use, as reported in other PLWHIV (Sohler et al., 2021), especially when treatment adherence is high (Adler et al., 2022). Further research is needed in this field, as a previous meta-analysis failed to identify specific moderators (Perera et al., 2017). Qualitative research in PLWHIV has described the role of family, partners, and providers (Sohler et al., 2021), which might help describe the patterns of meth use and non-adherence in our study population.

Participants without adherence had an increased risk of exchanging crystal meth for a sexual encounter. To the authors’ knowledge, no studies have investigated the specific relationship between crystal meth use in the context of transactional sex and non-adherence to HIV treatment. However, studies among gbMSM in Canada and the US–Mexico border show that there may be a significant association between crystal meth use and transactional sex (Armstrong et al., 2021; Loza et al., 2020). Additionally, previous research among gbMSM in California found an association between transactional sex (in exchange for money, substances, or shelter) and higher HIV viral load levels (Javanbakht et al., 2019). Finally, findings from in-depth interviews among a subsample of the same parent study indicate that some participants living with HIV were exchanging antiretrovirals for crystal meth on dating apps (Rafful et al., 2023). This exchange may compromise treatment adherence.

Interestingly, we found a significant association between risky cocaine use and non-adherence, and stigma. This may imply that participants who engage in more than one stimulant use have more complex conditions which may also affect their health. Previous studies have found that cocaine use was associated with faster HIV progression (Baum et al., 2009), and it may also be related to HIV stigma through the intersection of both stigmatized conditions (i.e., HIV-positive status and substance use) (Stringer et al., 2019). Further studies are needed to analyze this intersectionality in the Mexican setting.

This research presents several limitations. First, this study was based on self-report, including HIV status and non-adherence to HIV treatment. In addition, there is no gold standard to measure treatment adherence; however, we obtained similar data from other studies where adherence was assessed through viral load and other conditions related to ART adherence such as the pharmacy refill of ART (Sumari-de Boer et al., 2012). Second, because the sample was comprised of hardly reached persons and the conditions of the SARS CoV-2 health contingency, it was decided to use non-probabilistic web snowball sampling. Nevertheless, the sample belongs to key a population of the HIV epidemic: young adult gbMSM (CENSIDA, 2021b), which strengthens the importance of the present study. In addition, this sampling methodology has been recommended in health sciences due to the ethical implications of stopping research related to health phenomena such as substance use (Wang et al., 2021). Also, it has been observed that people tend to participate more sincerely in studies where they do not have to meet face-to-face with researchers (Evans et al., 2008). Third, the sample size was relatively small. However, persons who use crystal meth are a hardly reached population, and the inclusion criteria for this study also included other potentially stigmatized characteristics (i.e., being gbMSM and living with HIV). The results we obtained add to the existing knowledge on the intersectionality faced by gbMSM living with HIV and using crystal meth, which, to our knowledge, has rarely been addressed in Mexico. Fourth, considering that all participants used crystal meth and substance use is also a source of stigma, this probably influenced when reporting stigma. It is suggested that future studies consider the intersectionality between HIV-related stigma and substance use-related stigma to understand the impact they have on adherence to treatment.

### Conclusions

The findings of this study show the importance of considering the stigma in the implementation of interventions focused on promoting treatment adherence among gbMSM living with HIV. To achieve the “95–95–95” goals set out in 2030 (ONUSIDA, 2015) UNAIDS, national and local governments, and service providers may include more efforts addressing stigma among persons who use drugs. This study stresses the need for public health interventions to integrate HIV treatment services with non-judgmental stimulant use (Chan & Tang, 2021) and psychosocial services to promote treatment adherence among gbMSM who engage in substance use.

### Supplementary Information

Below is the link to the electronic supplementary material.

Supplementary file1 (DOCX 16 kb)

## Declarations

### Availability of Data and Materia

The data and materials supporting this study’s findings are available upon request.
