# Supplementary material for: HIV-Related Stigma and Treatment Adherence Among Gay, Bisexual, and Other Men Who Have Sex with Men Who Use Crystal Meth in the Metropolitan Area of Mexico City
Source: Arch Sex Behav. 2024 Feb 26;53(4):1561–74. doi: 10.1007/s10508-024-02816-6 (PMC10954905; doi:10.1007/s10508-024-02816-6)
Supplement: Supplementary file 1 — Supplementary file1 (DOCX 16 kb) [file 10508_2024_2816_MOESM1_ESM.docx]

| **Supplementay Table 1.** Correlation matrix to evaluate collinearity between variables associated with adherence to treatment. | | | | | | | | | | | |
| --- | --- | --- | --- | --- | --- | --- | --- | --- | --- | --- | --- |
|  | **Educational level** | **Health insurance** | **Risk associated with cocaine use** | **Risk associated with crystal meth use** | **Receiving crystal meth in exchange for a sexual encounter** | **Giving crystal meth in exchange for a sexual encounter** | **Evolution of the diagnosis from HIV to aids** | **Enacted stigma** | **Internalized stigma** | **Anticipated stigma** | **Total stigma** |
| Educational level | 1 |  |  |  |  |  |  |  |  |  |  |
| Health insurance | 0.096 | 1 |  |  |  |  |  |  |  |  |  |
| Risk associated with cocaine use | -0.05 | -0.39 | 1 |  |  |  |  |  |  |  |  |
| Risk associated with crystal meth use | -0.046 | -0.018 | 0.04 | 1 |  |  |  |  |  |  |  |
| Receiving crystal meth in exchange for a sexual encounter | -.285^**^ | -0.141 | 0.353 | -0.001 | 1 |  |  |  |  |  |  |
| Giving crystal meth in exchange for a sexual encounter | -0.093 | -0.203 | -0.221 | 0.138 | .398^**^ | 1 |  |  |  |  |  |
| Progression to aids | -.260^*^ | -0.026 | 0.123 | 0.136 | .287^**^ | -0.038 | 1 |  |  |  |  |
| Enacted stigma | -.217^*^ | -0.081 | 0.26 | 0.147 | .417^**^ | 0.179 | .240^*^ | 1 |  |  |  |
| Internalized stigma | -0.111 | -0.163 | **.543^**^** | .318^**^ | .313^**^ | 0.007 | 0.1 | .295^**^ | 1 |  |  |
| Anticipated stigma | -.291^**^ | -.232^*^ | 0.391 | .280^**^ | .420^**^ | 0.11 | .239^*^ | **.591^**^** | **.553^**^** | 1 |  |
| Total stigma | -.280^**^ | -0.202 | **.548^**^** | .324^**^ | .472^**^ | 0.114 | .225^*^ | **.749^**^** | **.816^**^** | **.884^**^** | 1 |
| * Significance at *p* < 0.05. ** Significance at *p* < 0.01.  Correlations at *r* > 0.5 are bolded. | | | | | | | | | | | |
